# Fabrication of Vertical-Standing Co-MOF Nanoarrays with 2D Parallelogram-like Morphology for Aqueous Asymmetric Electrochemical Capacitors

**DOI:** 10.3390/molecules26175394

**Published:** 2021-09-05

**Authors:** Leyuan Li, Hongtian Mi, Yuhong Jin, Dayong Ren, Kailing Zhou, Qianqian Zhang, Jingbing Liu, Hao Wang

**Affiliations:** Key Laboratory for New Functional Materials of Ministry of Education, Institution of Advanced Energy Materials and Devices, Faculty of Materials and Manufacturing, Beijing University of Technology, Beijing 100124, China; Lileyuan@emails.bjut.edu.cn (L.L.); ustbmht@163.com (H.M.); rendayong1@emails.bjut.edu.cn (D.R.); zkling@emails.bjut.edu.cn (K.Z.); zhangqianqian@bjut.edu.cn (Q.Z.); liujingbing@bjut.edu.cn (J.L.)

**Keywords:** metal organic frameworks, Co-MOF, nanoarrays, asymmetric electrochemical capacitors

## Abstract

Metal organic frameworks (MOFs) have been considered as one of the most promising electrode materials for electrochemical capacitors due to their large specific surface area and abundant pore structure. Herein, we report a Co-MOF electrode with a vertical-standing 2D parallelogram-like nanoarray structure on a Ni foam substrate via a one-step solvothermal method. The as-prepared Co-MOF on a Ni foam electrode delivered a high area-specific capacitance of 582.0 mC cm^−2^ at a current density of 2 mA cm^−2^ and a good performance rate of 350.0 mC cm^−2^ at 50 mA cm^−2^. Moreover, an asymmetric electrochemical capacitor (AEC) device (Co-MOF on Ni foam//AC) was assembled by using the as-prepared Co-MOF on a Ni foam as the cathode and a active carbon-coated Ni foam as the anode to achieve a maximum energy density of 0.082 mW cm^−2^ at a power density of 0.8 mW cm^−2^, which still maintained 0.065 mW cm^−2^ at a high power density of 11.94 mW cm^−2^. Meanwhile, our assembled device exhibited an excellent cycling stability with a capacitance retention of nearly 100% after 1000 cycles. Therefore, this work provides a simple method to prepare MOF-based material for the application of energy storage and conversion.

## 1. Introduction

Nowadays, in order to fight climate change and promote green, low-carbon development, developing renewable energy sources, such as wind energy, solar energy, geothermal energy, tidal energy, has been considered as one of the most promising and effective ways to decrease the greenhouse gas emission [1,2,3]. However, these renewable energy sources are intermittent and uncontrollable. Therefore, it is very urgent to explore a reliable electrochemical energy storage device to efficiently store these renewable energy sources. Among these electrochemical energy storage devices, electrochemical capacitors have drawn remarkable attention owing to their high power density and long cycling stability along with fast energy storage ability [4,5,6,7]. According to the mechanism of energy storage, electrochemical capacitors are classified into electric double-layer capacitors (EDLCs) with the physical adsorption of ions and pseudocapacitors with the electron transfer reaction. EDLCs that usually use carbon-based electrode materials display high power density and excellent cycling performance but deliver a low capacitance. In contrast, pseudocapacitors have the high energy density with the poor cyclic stability and unsatisfied power density [8,9]. Therefore, it is of great importance to improve the energy density of the electrochemical capacitors accompanied with the high power density and long cycling stability.

Recently, asymmetric electrochemical capacitors (AECs) have been designed as a new single-energy storage device, which integrates a high battery-like energy density electrode and an EDLC electrode [10,11,12]. Due to the utilization of the battery-like electrode and the wide working voltage range, AECs can simultaneously improve the energy/power density [13]. Thus, it should be noted that the study on the battery-like electrode materials plays a crucial role for high-performance AECs.

Compared with the traditional battery-like electrode materials, such as transition metal oxides [14,15], sulfides [16], hydroxides [17], phosphides [18,19,20], selenides [21,22], and even conducting polymers [23,24,25], metal organic frameworks (MOFs) have become a research hotspot for the application of electrochemical capacitors due to their large specific surface area, abundant pore structure, and different active transition metal ions including Fe, Co, Cu, and Ni [26,27,28,29]. So far, it is very popular to take advantage of MOFs as a template to prepare different derivatives via the high-temperature treatment process because it can be easy to obtain carbon-coated active transition metal-based composites [30,31,32]. Nevertheless, the framework structure of MOFs is inevitably destroyed during the pyrolytic process, which can significantly reduce the specific surface area and redox active sites. Thus, it can be expected that the advantage of the pristine MOFs may be achieved by the direct application as an electrode material for electrochemical capacitors. However, pristine MOFs exhibit low intrinsic electrical conductivity, which limits their application in electrochemical capacitors.

In order to deal with this problem, Dinca’s group [33] designed a conductive pristine MOF from the regulation of the molecular structure for super capacitive applications. A MOF Ni_3_(2,3,6,7,10,11-hexaiminotriphenylene)_2_ (Ni_3_(HITP)_2_) with high electrical conductivity and high porosity displayed a specific area capacitance of 18 μF cm^−2^ and good capacity retention of 90% for 10,000 cycles. It could be found that Ni_3_(HITP)_2_ exhibited a EDLC mechanism with a low capacitance. Another strategy used to improve the electrical conductivity of MOFs is combined with different carbon materials. Azadfalah and coworkers [34] reported a Co-based MOF with graphene (CoMG) nanocomposite via a one-step method. The CoMG nanocomposite delivered a high specific capacitance of 549.96 F g^−1^ at 1.0 A g^−1^. The asymmetric-activated carbon–CoMG device exhibited a specific energy of 8.1 Wh kg^−1^ at 850 W kg^−1^ with a long cycle life of 78.85% capacitance retention after 1000 cycles at 1 A g^−1^, yet the addition of inactive carbon (including in the composite and conductive carbon black) and binder for the assembly of an electrode on the current collector would decrease the capacitance value of the as-prepared composites. Recently, Xu and coworkers [35] designed a hierarchical layered structure of CoNi-MOF with ultrathin nanosheets and nanotube arrays on a carbon cloth (CC/CoNi-MOF). ZnO nanorods, as a sacrificial template, were firstly deposited on the CC, and then Co(OH)_2_ nanosheets were coated on ZnO nanorods via an electrodeposition method. Finally, Co(OH)_2_ was thoroughly changed into CoNi-MOF in the nickel nitrate hexahydrate and p-benzenedicarboxylic as an organic links system via a hydrothermal process. As-prepared CC/CoNi-MOF exhibited a high areal capacity of 1.01 C cm^−2^ at a current density of 2 mA cm^−2^ with a high energy density of 55.5 Wh kg^−1^ at 175.5 W kg^−1^ for the AEC device. The good electrochemical capacitive performance could be attributed to the intimate contact between CoNi-MOF, CC, and the 2D nanosheet-like structure, which could enhance the electron and ion transportation.

Inspired by this work, in order to design a simple method for the preparation of 2D MOF arrays on the conductive substrate, we developed a Co-MOF electrode with vertical-standing 2D parallelogram-like nanoarray structure on a Ni foam substrate via a one-step solvothermal method. As-prepared Co-MOF on a Ni foam electrode delivered a large area specific capacitance of 582.0 mC cm^−2^ at a 2 mA cm^−2^, while maintaining a 60.1% capacity retention at 50 mA cm^−2^. Moreover, as for AES device, a maximum energy density was as large as 0.082 mW cm^−2^ at a power density of 0.8 mW cm^−2^ along with an excellent cycling stability (a capacitance retention of nearly 100% after 1000 cycles).

## 2. Results and Discussion

**Figure 1:**
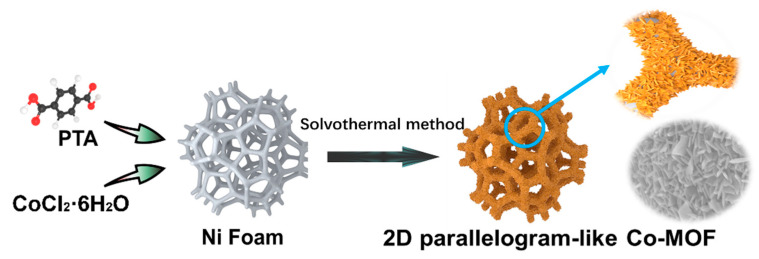
Schematic diagram of the synthetic process for 2D parallelogram-like Co-MOF on Ni foam.

Figure 1 exhibits the synthetic process for 2D parallelogram-like Co-MOF on Ni foam through one-step solvothermal method. Briefly, CoCl_2_·6H_2_O and PTA were completely dispersed into a mixed solvent of DMF and high-purity water. During the reaction process in the autoclave at 120 °C, the color of Ni foam went from the original silver-gray to light-brown, which means that the Co-MOF material was grown on the surface of Ni foam.

### 2.1. Structure Characterization of As-Prepared Co-MOF on Ni Foam

**Figure 2:**
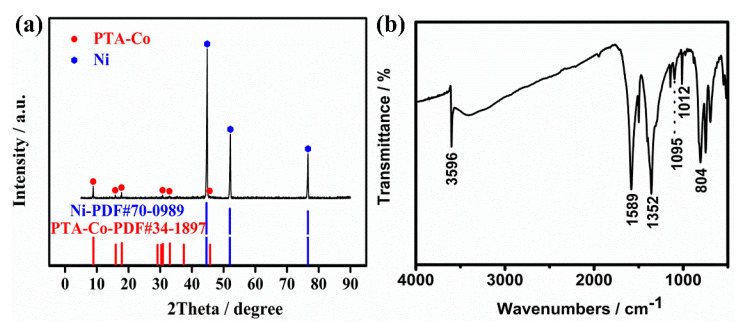
(**a**) XRD pattern and (**b**) FT-IR spectra of 2D parallelogram-like Co-MOF on Ni foam.

Figure 2 exhibits the XRD pattern of as-prepared 2D parallelogram-like Co-MOF on Ni foam. The diffraction peaks at 44.8°, 52.0°, and 76.5° are attributed to the nickel (PDF#70-0989) from the Ni foam substrate. Meanwhile, it can be observed that the 2θ values of the peaks at 8.8°, 15.8°, 17.9°, 30.7°, 32.8°, and 45.7° belong to the cobalt terephthalate hydroxide (Co_2_(OH)_2_(C_8_H_4_O_4_)) phase, in agreement with the simulated crystal data from PDF#34-1897 [36,37]. These results confirm that Co-MOF was successfully deposited on Ni foam via a simple solvothermal method. In addition, the organic functional groups of as-prepared 2D parallelogram-like Co-MOF are confirmed by the FT-IR spectrum, as shown in Figure 2b. The absorption band at 3596 cm^−1^ is assigned to the O-H stretching vibration, while the two main bands at 1589 and 1352 cm^−1^ are attributed to antisymmetric and symmetric -COO functional groups, respectively [38,39,40]. Another three absorption bands at 1095, 1012, and 804 cm^−1^ correspond to C-H vibration in aromatic rings [36,41].

**Figure 3:**
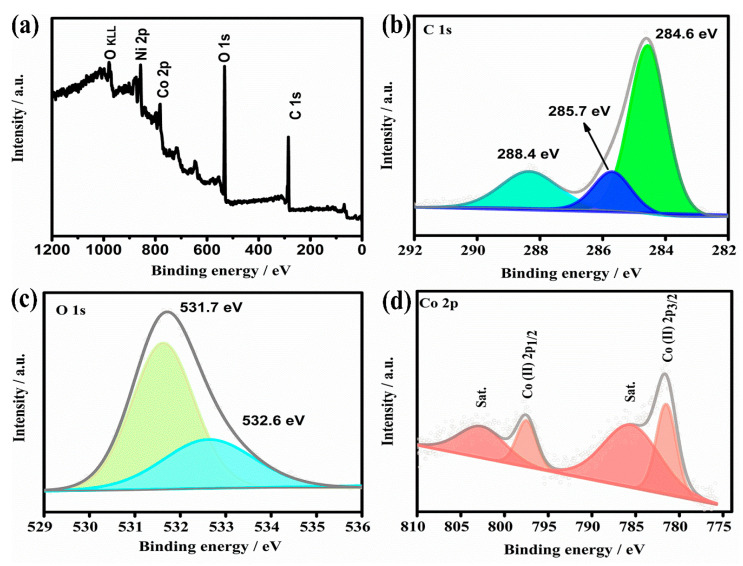
(**a**) XPS survey spectrum, (**b**) C 1s, (**c**) O 1s and (**d**) Co 2p region of 2D parallelogram-like Co-MOF on Ni foam.

XPS measurements are also used to further confirm the surface elemental states of as-prepared 2D parallelogram-like Co-MOF on Ni foam. As shown in Figure 3a, XPS survey spectrum exhibits Co, Ni, C, and O peaks. The existence of Ni peak is attributed to the Ni substrate. Figure 3b shows the high-resolution XPS spectrum of C 1s. It can be resolved into three peaks at binding energies of 284.6, 285.7, and 288.4 eV, which can be ascribed to C=C, C-H and O-C=O groups for PTA ligand at Co-MOF materials [42]. Meanwhile, the high-resolution XPS spectrum of O 1s is shown in Figure 3c. The peaks at 531.7 and 532.6 eV are identified as the coordination between the O-Co and C=O groups in PTA ligands, respectively. For the high-resolution XPS spectrum of Co 2p, two main peaks at binding energies of 797.6 and 781.4 eV, accompanied with two shake up satellites at 802.9 and 785.9 eV for Co 2p1/2 and Co 2p3/2 (names as “Sat.”), respectively, can be observed [43]. It is well known that the binding energy gap between the main peak and the satellite peak is considered as an important parameter to confirm the oxidation of transition metals [44]. As for the Co (II), the energy gap is within 6 eV. Based on these analyses, we can conclude that the chemical valence of Co ions in our 2D parallelogram-like Co-MOF on Ni foam is predominantly 2+.

### 2.2. Morphological Characterization of As-Prepared Co-MOF on Ni Foam

**Figure 4:**
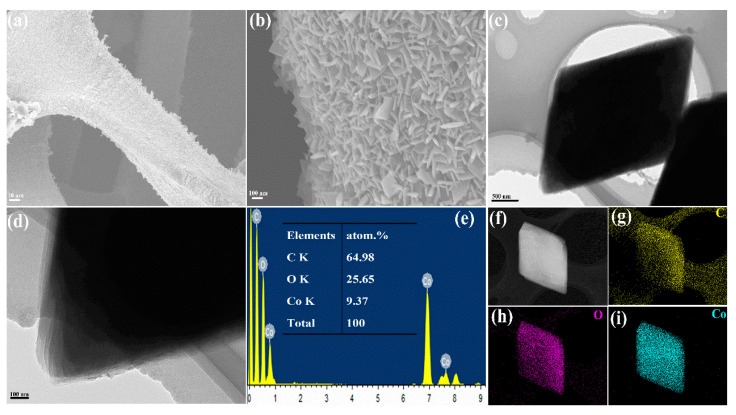
(**a**,**b**) FESEM images, (**c**,**d**) TEM images, (**e**) EDX spectrum, and (**f**–**i**) the corresponding elemental mapping images of C, O, and Co for 2D parallelogram-like Co-MOF on Ni foam.

The morphology of the as-prepared sample produced by the solovothermal process was obtained by FE-SEM. It can be clearly observed in Figure 4a that the surface of Ni foam becomes rough compared with the bared Ni foam (Inset of Figure 4a), which is attributed to the formation of Co-MOF on Ni foam. More interestingly, as shown in Figure 4b, the high-density vertical-standing nanoarrays of Co-MOF with a 2D nanosheet morphology are fully covered on the surface of Ni foam. Furthermore, as we can see from the TEM image (Figure 4c), the 2D nanosheet morphology of as-prepared Co-MOF on Ni foam displays a unique parallelogram. Moreover, in Figure 4d, it can be found that 2D parallelogram-like Co-MOF is a layered structure. The EDX spectrum (Figure 4e) of as-prepared 2D Co-MOF exhibits the atom ratios of C, O, and Co elements, which are 64.98, 25.65 and 9.37%, respectively. The corresponding elemental mapping images are shown in Figure 4f–i. It demonstrates that the C, O, and Co elements are uniformly distributed in our 2D parallelogram-like Co-MOF.

### 2.3. Electrochemical Capacitive Perforamcne of As-Prepared Co-MOF on Ni Foam

**Figure 5:**
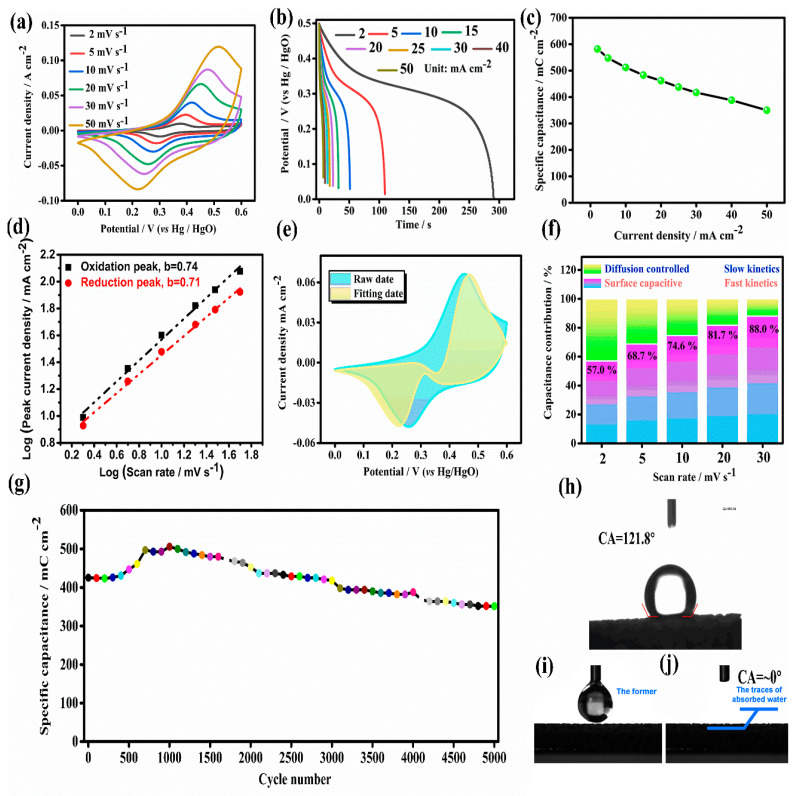
Electrochemical capacitive performance of 2D parallelogram-like Co-MOF on Ni foam using a classical three-electrode system: (**a**) CV curves at different scan rates, (**b**) GCD curves at different current densities, (**c**) Specific area capacitance at a current density range from 2 to 50 mA cm^−2^, (**d**) Plots between Log (scan rate) and Log (peak current density), (**e**) Capacitive and diffusion currents at 20 mV s^−1^, (**f**) Contribution ratio of pseudocapacitive and diffusion-controlled capacitance, (**g**) Cyclic stability at a current density of 20 mA cm^−2^ and the contact angle test of (**h**) bared Ni foam and (**i**,**j**) as-prepared Co-MOF on Ni foam.

Electrochemical capacitive performance of 2D parallelogram-like Co-MOF on Ni foam was studied by using a classical three-electrode system. As shown in Figure 5a, CV curves of as-prepared 2D MOF on Ni foam are obtained at different scan rates of 2, 5, 10, 20, 30, and 50 mV s^−1^ with a potential range of 0–0.6 V. It can be observed that the shapes of CV curves display a clear anodic and cathodic peak at ~0.25 and ~0.45 V, respectively, which may be attributed to the redox reaction of Co^2+^/Co^3+^ in Co-MOF accompanied with the action of OH- during the electrochemical capacitive process [45,46]. Furthermore, the anodic and cathodic peaks can also be found at a high scan rate of 50 mV s^−1^, indicating the good reversibility of our 2D parallelogram-like Co-MOF on Ni foam for the fast charge–charge process. Figure 5b exhibits the discharge curves of 2D MOF on Ni foam at different current densities (from 2 to 50 mA cm^−2^) under a potential range of 0–0.5 V. It can be found that an obvious slope change (0.32–0.45 V) appears in the discharge curves, which agrees well with the CV results. The maximum areal capacitance values of 2D parallelogram-like Co-MOF on Ni foam can be reached at 582.0 mC cm^−2^ at a current density of 2 mA cm^−2^. Meanwhile, even at a very large current density of 50 mA cm^−2^, the capacitance value of as-prepared 2D Co-MOF electrode can still be 350.0 mC cm^−2^, indicating the good rate performance of 60.1% (as shown in Figure 5c). A comparison between the reported results and our current study (based on active material mass: 1.23 mg cm^−2^ for our Co-MOF) is displayed in Table 1. It can be seen that our Co-MOF electrode materials exhibit better electrochemical capacitive performance.

The electrochemical behavior of 2D parallelogram-like Co-MOF on Ni foam is further studied by fitting the CV curves. The following equation is used to fit the scan rate (*v*, mV s^−1^) and peak current (*I_p_*, mA cm^−2^) from CV curves [49,50,51].

(1) $$I_{p}=av^{b}$$

(2) $$Log(I_{p})=bLog(v)+Log(a)$$

The slope *b* for the relationship between *Log*(*Ip*) and *Log*(*v*) is a constant, which can disclose the electrochemical charge storage mechanism for as-studied active electrode materials. When *b* value equals to 0.5, it means that the electrode current response is controlled by a diffusion process. When b value equals to 1, it is reprehensive of a capacitive-controlled response. When *b* value is between 0.5 and 1, the capacitance contribution is attributed to the surface capacitive and diffusion processes. Figure 5d exhibits the fitting *b* values of 0.71 and 0.74 for the anode and cathode, respectively, suggesting that as-prepared 2D Co-MOF electrode displays the electrode reaction controlled by surface and diffusion processes. In order to further differentiate the contribution rate of the diffusion process and surface capacitive-controlled reaction, the following equation can be used [52,53].

(3) $$i(V)=k_{1}v+k_{2}v^{1/2}$$

Figure 5e displays the CV curve at 20 mV s^−1^, and the capacitive contribution ratios are 57.0%, 68.7%, 74.6%, 81.7%, and 88.0% (Figure 5f) at 2, 5, 10, 20, and 30 mV s^−1^, respectively. It can be observed that the surface capacitive response rises with the increased scan rate of CV, indicating the diffusion process was delayed at a high scan rate. Therefore, we can conclude that the electrochemical charge storage process for our 2D parallelogram-like Co-MOF on a Ni foam electrode is controlled by a mixture of capacitor and battery-type properties. Cyclic stability of as-prepared 2D parallelogram-like Co-MOF on Ni foam is also tested at a high current density of 20 mA cm^−2^. It can be observed that the initial capacitance value is 425.5 mC cm^−2^ along with the increased capacity after 1000 cycles, which can be attributed to the adequate activation process. Then, the capacitance retention can be 82.6% compared with the initial capacitance value after 5000 cycles, indicating its good cyclic stability.

Based on the above-mentioned results, the good electrochemical capacitive performance for our 2D parallelogram-like Co-MOF on Ni foam may be attributed to the following reasons: (1) directly grown Co-MOF on the surface of conductive Ni foam can guarantee the good electrical conductivity, which can help the electron transportation; (2) this self-supported nanostructure design can maximize active material utilization (Co-MOF) without the polymer binder and conductive agents; (3) vertical standing Co-MOF nanoarrays with a 2D parallelogram-like morphology can provide abundant active surface areas to react with electrolyte ions; and (4) super-hydrophilic property of Co-MOF on Ni foam (as shown in Figure 5h,i) is advantageous to contact with the aqueous electrolyte.

### 2.4. Electrochemical Perforamcne of AEC Device

**Figure 6:**
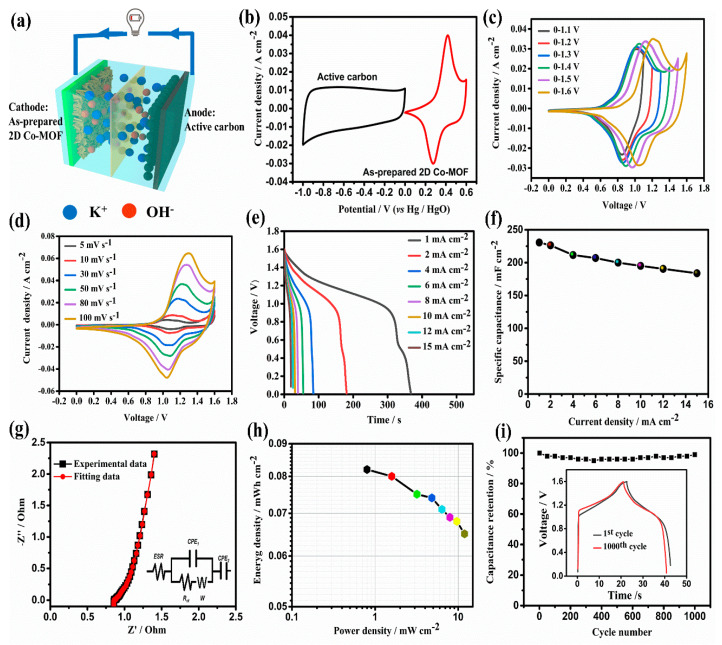
Electrochemical performance of 2D parallelogram-like Co-MOF on Ni foam//AC AEC device: (**a**) Illustration of asymmetric supercapacitor device using as-prepared 2D parallelogram-like Co-MOF on Ni foam and AC as a cathode and anode material, respectively, (**b**) 2D parallelogram-like Co-MOF and AC at the different potential windows at 10 mV s^−1^, (**c**) CV curves at a scan rate of 50 mV s^−1^ with different voltage ranges, (**d**) CV curves at different scan rates at a voltage range from 0–1.6 V, (**e**) Discharge curves at different current densities, (**f**) Specific capacitance varied from current densities, (**g**) EIS plots for experimental and fitting data, (**h**) Ragone plots of energy and power density, and (**i**) Cyclic performance at a current density of 15 mA cm^−2^, inset shows GCD curves for the first and 1000th cycle.

As shown in Figure 6a, an AEC device was assembled with our 2D parallelogram-like Co-MOF on Ni foam as a cathode material and active carbon coated on Ni foam as an anode material, and electrochemical performance was tested in a 2 M KOH electrolyte. The CV curves of as-prepared Co-MOF cathode and active carbon anode are obtained at a scan rate of 10 mV s^−1^, as shown in Figure 6b. It can be found that the potential range of as-prepared Co-MOF cathode is from 0 to 0.6 V, while the active carbon anode is −1 to 0 V. Therefore, it can be expected that the suitable working voltage of our assembled AEC device can reach up to 1.6 V, which can be further confirmed by CV curves at a scan rate of 50 mV s^−1^ with different voltage ranges obtained from Figure 6c. Subsequently, CV curves at different scan rate rage from 5 to 100 mV s^−1^ at a voltage range from 0–1.6 V are shown in Figure 6d. It can be observed that the shape of all CV curves almost keep unchanged with the increased scan rates, even at a high scan rate of 100 mV s^−1^, indicating the excellent performance rate of our assembled AEC device. Figure 6e exhibits the discharge curve for the device at a current density range from 1 to 15 mA cm^−2^. The areal specific capacitance values (Figure 6f) determined by the charge curves are 230.6, 226.3, 211.5, 207.0, 200.0, 195.0, 190.5, and 183.8 mF cm^−2^ at 1–15 mA cm^−2^, respectively. The high capacitance retention of 79.7% further supports the excellent capacitive characteristic for our Co-MOF-based AEC device. EIS measurement and the fitting results based on the equivalent circuit (Figure 6g, inset) for the AEC device are shown in Figure 6g. It can be seen that the plot display a small semi-circle at the high-frequency region and a nearly vertical line at the low-frequency region. The fitting data show that the equivalent series resistance (ESR) value is 0.89 along with a small charge transfer resistance (Rct) of 0.02. These analyzes demonstrate that our AEC device displays good super-capacitive behavior. Especially in Ragone plots (Figure 6h) of energy and power density for our AEC device, the maximum energy density can reach up to 0.082 mW cm^−2^ with a power density of 0.8 mW cm^−2^. Moreover, the energy density can still maintain 0.065 mW cm^−2^ at a high power density of 11.94 mW cm^−2^.

The cyclic performance of our AEC device is also tested at a current density of 15 mA cm^−2^ for 1000 cycles. It shows a capacitance retention of nearly 100% after 1000 cycles. The GCD curves for the initial and the 1000th cycle are almost overlapped, which further confirms the excellent cycling stability of our Co-MOF-based electrode materials. These results are comparable or higher to many reported pristine MOF-based electro-capacitive materials [54,55].

## 3. Materials and Methods

### 3.1. Materials

p-phthalic acid (PTA) was purchased from Energy Chemical Co, Ltd. (Shanghai, China). Nickel chloride hexahydrate (CoCl_2_·6H_2_O), hydrochloric acid (HCl), N, N-dimethylformamide (DMF), and methanol were purchased from Tianjin Fuchen Chemical Reagent Co., Ltd. Nickel foam (surface density: 350 g m^−2^; thickness: 1.6 mm) was brought from Shenzheng Kejing Star Technology Company (Shengzheng, China).

### 3.2. Preparation of Co-MOF on Ni Foam

In a typical experiment, 2.0 mmol of CoCl_2_·6H_2_O and 2.0 mmol of PTA were dissolved in 40 mL of DMF. After stirring for 30 min at room temperature, 2.0 mL of high-purity water was slowly dropped into above-mentioned solution and stirred for 10 min. Next, the solution was transferred into a Teflon-lined stainless steel autoclave. At the same time, the pretreated Ni foam (1 × 3 cm^2^) was immediately immersed into an autoclave. After that, the autoclave was placed into an oven and maintained at a temperature of 120 °C for 180 min. After that, as-prepared Co-MOF on Ni foam was obtained by washing with DMF and methanol for at least three times. Finally, as-prepared samples were dried at 80 °C for 12 h in the vacuum drying oven.

### 3.3. Physical Characterization

The crystalline structure of as-prepared samples was characterized by X-ray diffraction (XRD) pattern carried out using a Bruker D8-Advanced X-ray instrument (Karlsruhe, Germany) with Cu-K α radiation (λ = 1.540598 Å). Fourier transform infrared (FTIR) spectroscopy was collected on a FTIR-650 spectrophotometer (Tianjin Gangdong Co., Ltd., Tianjin, China). The microstructure and morphology were observed by a field-emission scanning electron microscope (FESEM, Hitachi S4800, Tokyo, Japan) and transmission electron microscopy (TEM, FEI Tecnai G2 F30, Hillsboro, OR, USA). X-ray photoelectron spectrometer (XPS) was performed using Thermo Fisher ESCALAB 250 Xi (Waltham, MA, USA).

### 3.4. Electrochemical Measurements

Electrochemical measurements were carried out on a CHI660e electrochemical workstation (Shanghai CH Instruments Ins., Shanghai, China) in a classical three-electrode, along with as-prepared Co-MOF on Ni foam as the work electrode, a Pt plate as the counter electrode, and an Hg/HgO as the reference electrode. Next, 2 M KOH was chosen to be the electrolyte. Cyclic voltammetry (CV) measurements were displayed at different scan rate (2–50 mV s^−1^) with a potential range from 0 to 0.6 V. Galvanostatic charge discharge (GCD) curves were performed at different current densities (2–50 mA cm^−2^) with a potential range from 0 to 0.5 V.

The area specific capacitance (*Cs*) of as-prepared active Co-MOF on a Ni foam electrode was calculated by the following equation:

(4) $$C_{s}=\frac{I\times \Delta t}{s}$$

where *I*, Δ*t*, and *s* represent the discharge current (A), discharge time (s), and the area (cm^2^) of the electrode, respectively.

The home-made ACS device was assembled by using as-prepared Co-MOF on Ni foam as the cathode and active carbon-coated Ni foam as the anode, with a cellulosic separator in between. The mass ratio of cathode to anode was determined according to the charge balance equation. The area energy density *E* (Wh cm^−2^) and power density *P* (W cm^−2^) were calculated by the following equations:

(5) $$E=\frac{C\Delta V^{2}}{2}\times \frac{1000}{3600}$$

(6) $$P=\frac{E}{\Delta t}\times 3600$$

## 4. Conclusions

In summary, vertical-standing Co-MOF nanoarrays with 2D parallelogram-like morphology on Ni foam were successfully fabricated via a simple one-step solovothermal method. The as-prepared Co-MOF on a Ni foam electrode displayed good electrochemical capacitive performance due to the self-supported nanostructure design, the intimate contact between active Co-MOF and conductive Ni foam, and super-hydrophilic property of Co-MOF on Ni foam. The practicality of our Co-MOF on Ni foam electrodes were confirmed by the assembled AEC device, which can achieve a maximum energy density of 0.082 mW cm^−2^ at a power density of 0.8 mW cm^−2^ with a capacitance retention of nearly 100% after 1000 cycles. This work will open up a simple method for preparing high-performance MOF-based energy storage materials.

## Figures and Tables

**Table 1:** A comparison of electrochemical capacitive performance for pristine MOF-based materials between the reported results and our current study (Based on active material mass: 1.23 mg cm^−2^ for our Co-MOF).

Electrode	Synthetic Method	Morphology	Electrolyte	Capacitance/Capacity	Reference
NiCo-MOF	Chemical method	Nanosheets	2 M KOH	1109 F g^−1^ at 0.5 A g^−1^	[26]
Mn-MOF	Hydrothermal	Crystalline	6 M KOH	433 F g^−1^ at 1 A g^−1^	[28]
Co-MOF@Graphene	Ultrasonicate and centrifugation	Nanoparticles	6 M KOH	549.96 F g^−1^ at 10 mV s^−1^	[36]
Co-MOF	Hydrothermal	Nanospheres	1 M NaOH	657 F g^−1^ at 0.5 A g^−1^	[40]
Co-MOF	Hydrothermal	Microspheres	1 M LiOH	553 F g^−1^ at 1 A g^−1^	[42]
Co-doped Ni-based MOF	Hydrothermal	Nanoflowers	6 M KOH	100 F g^−1^ at 1 A g^−1^	[44]
Mo&polyoxometalate-MOF	Hydrothermal	2D layered microstructure	1 M H_2_SO_4_	249.0 F g^−1^ at 3 A g^−1^	[47]
MOF-5	Incipient wetness technique	Nanoparticles	1 M H_2_SO_4_	100 F g^−1^ at 5 mV s^−1^	[48]
Co-MOF	Hydrothermal	nanoarrays	2 M KOH	946 F g^−1^ at 1.63 A g^−1^ 582.0 mC cm^−2^ at 2 mA cm^−2^	This Work

## Data Availability

Data available on request.

## Sample Availability

Samples of the compounds are unavailable from the authors.
